# The Role of APOL1 in Necrotizing Enterocolitis and Its Promise as a Diagnostic Biomarker

**DOI:** 10.1155/mi/8637617

**Published:** 2026-02-24

**Authors:** Jie-Ting Lu, Qiu-Hua Wang, Ying-Yan Liu, Song Tian, Long-Long Hou, Xin Zhong, Li-Zhu Chen, Qian Zhang, Peng-Fei Wei, Lin Li, Yan Tian, Qiu-Ming He, Yu-Feng Liu, Gen-Quan Yin, Yu Ouyang, Lin Liao, Wei Zhong, Chao-Ting Lan

**Affiliations:** ^1^ Department of Pediatric Surgery, Guangzhou Women and Children’s Medical Center, Liuzhou Hospital, Liuzhou, Guangxi, China, gzfezx.com; ^2^ The First School of Clinical Medicine, Southern Medical University, Guangzhou, Guangdong, China, fimmu.com; ^3^ Department of Clinical Laboratory, The First Affiliated Hospital of Guangxi Medical University, Key Laboratory of Clinical Laboratory Medicine of Guangxi Medical University, Education Department of Guangxi Zhuang Autonomous Region, Nanning, Guangxi, China, gxmu.edu.cn; ^4^ Center for Medical Research on Innovation and Translation, Guangzhou First People’s Hospital, The Second Affiliated Hospital of South China University of Technology, Guangzhou, Guangdong, China, gzhosp.cn; ^5^ Department of Neonatal Surgery, Guangdong Women and Children Hospital, Guangzhou, Guangdong, China, e3861.com; ^6^ Department of Pediatric Surgery, Guangzhou Women and Children’s Medical Center, Guangzhou Medical University, Guangzhou, Guangdong, China, gzhmc.edu.cn; ^7^ Department of Pediatrics, Nanfang Hospital, Southern Medical University, Guangzhou, Guangdong, China, fimmu.com; ^8^ Department of Anesthesiology, Jiangxi Provincial Children’s Hospital, Nanchang Medical College, JXHC Key Laboratory of Jiangxi Provincial Children’s Hospital, Nanchang, Jiangxi, China, jxsetyy.cn

**Keywords:** APOL1, diagnosis, immunity, inflammation, macrophage

## Abstract

**Objective:**

Necrotizing enterocolitis (NEC) is a severe inflammatory disease of the intestine. Although previous studies have demonstrated that APOL1 plays an important role in regulating macrophage polarization and immune‐mediated inflammatory diseases, its specific function in NEC remains unclear. We aim to further explore the role of APOL1 in NEC and its efficacy as a diagnostic biomarker.

**Methods:**

We performed tissue transcriptomic and plasma proteomic analyses based on clinical samples from preterm infants with NEC patients and conducted multilevel experimental validations. An in vitro macrophage polarization model was established to further investigate the regulatory role of APOL1 in macrophage differentiation. The diagnostic potential of APOL1 for NEC was evaluated using receiver operating characteristic (ROC) curve analysis.

**Results:**

Multiomics analyses revealed a significant upregulation of APOL1 expression in NEC, which was further confirmed by qPCR, immunofluorescence, and ELISA. Pathway enrichment and immune infiltration analyses indicated that APOL1 is positively associated with M1 macrophage infiltration and the expression of multiple proinflammatory cytokines. Immunofluorescence staining further demonstrated that APOL1 is highly expressed in M1 macrophage‐enriched regions. In vitro experiments showed elevated APOL1 expression in M1 macrophages, while its inhibition significantly reduced intracellular reactive oxygen species (ROS) accumulation and NF‐κB p65 activation, thereby suppressing M1 polarization. Moreover, the combination of plasma APOL1 and lymphocyte count (LYM) demonstrated high diagnostic efficacy for NEC, with an AUC value of 0.96 (sensitivity 93.3% and specificity 93.3%).

**Conclusions:**

Our findings reveal a marked upregulation of APOL1 in preterm infants with NEC. Mechanistically, we propose that the APOL1‐ROS‐NF‐κB axis constitutes a novel and promising therapeutic target for NEC intervention. Furthermore, the combined detection of plasma APOL1 and LYM demonstrates high efficacy.

## 1. Introduction

Necrotizing enterocolitis (NEC) is a severe intestinal inflammatory disease predominantly affecting preterm infants, with an estimated incidence of 7.0%–10% in very low birth weight infants [1] and a mean mortality rate of 20%–30% [2, 3]. The exact pathogenesis of NEC remains unclear. However, the immune inflammatory response has been confirmed as a key pathophysiological mechanism, with macrophage polarization playing a crucial regulatory role in the disease [4, 5]. The occurrence of NEC is closely related to multiple factors, including intestinal immaturity due to prematurity, dysbiosis, systemic inflammatory response, hypoxic‐ischemic injury, and genetic susceptibility. These risk factors can contribute to the development and progression of NEC by regulating macrophage polarization [6–8]. Apolipoprotein L1 (APOL1) reportedly regulates macrophage polarization balance (M1/M2 phenotype conversion) and contributes to immune‐mediated disease damage [9, 10]. Our preliminary study found that the expression of macrophage regulatory molecule APOL1 was significantly upregulated in NEC patient tissues and peripheral blood, yet its role in NEC remains unreported. Given the clinical challenge of insufficient sensitivity and specificity of current NEC diagnostic markers, this study aims to explore the molecular mechanisms of APOL1 in NEC pathogenesis and its diagnostic potential.

APOL1 is a protein predominantly synthesized and secreted into the bloodstream by the liver and is an important member of the apolipoprotein L family [11]. Recent studies have identified its crucial role in innate immunity and macrophage regulation. In vitro experiments have confirmed that APOL1 can induce the differentiation of THP‐1 monocytes into M1 macrophages and significantly upregulate the expression levels of proinflammatory cytokines such as IL‐6 and TNF‐α. This mechanism may play a significant role in the immune‐inflammatory damage associated with various diseases [10]. In ulcerative colitis, APOL1 overexpression in fibroblasts induces pyroptosis via the NLRP3/Caspase‐1/GSDMD pathway and releases inflammatory cytokines (IL‐6, IL‐1β, and TNF‐α) and chemokines (CXCL1, CXCL2, and CXCL9) [12]. Similarly, in inflammation‐induced β‐cell damage models, elevated APOL1 expression correlates with increased cell death and inflammation [13].

This study for the first time reveals that APOL1 can serve as a novel diagnostic biomarker for NEC. It may drive NEC progression by enhancing M1 macrophage polarization and proinflammatory cytokine release through reactive oxygen species (ROS)–dependent activation of the NF‐κB signaling pathway. This finding offers a new therapeutic target for the diagnosis and treatment of NEC.

## 2. Materials and Methods

### 2.1. Study Subjects

Our study included two groups: preterm infants with NEC (gestational age < 34 weeks) and controls diagnosed with intestinal atresia or other noninflammatory, nongastrointestinal diseases. Inclusion and exclusion criteria were as follows:

NEC group:

Patients with confirmed NEC (Bell’s stage II or higher) [14].
No concurrent autoimmune or congenital diseases.

Control (CTRL) group:

Patients with intestinal atresia or other noninflammatory, nongastrointestinal diseases.
No concurrent autoimmune or congenital diseases.
Not in the acute phase of enteritis or other inflammatory diseases.

Participants in both groups were matched for gestational age, weight, and sex, strictly adhering to the inclusion criteria.

Clinical samples were sourced from the repository at the Guangzhou Women and Children’s Medical Center.

### 2.2. RNA Sequencing

Total RNA was extracted from ileal tissues of Surgical NEC (*n* = 7) and CTRL (*n* = 7) using the TRIzol method. After RNA quality assessment, mRNA was purified, fragmented, and used for cDNA synthesis with random primers. Sequencing libraries were then constructed and sequenced on the Illumina HiSeq NovaSeq 6000 platform.

### 2.3. Plasma Proteomics

Plasma samples from 27 NEC (Medical NEC = 10 and Surgical NEC = 17) patients and 20 CTRL patients were processed by centrifugation, protein quantification, SDS‐PAGE separation, and staining, with a pooled sample used for quality control. After quality control, samples were reduced with DTT, blocked with IAA, and digested overnight with trypsin. Peptides were analyzed using an Orbitrap Astral mass spectrometer coupled to a Vanquish Neo system (Thermo Scientific) in DIA mode. DIA data were processed with DIA‐NN 1.8.1.

### 2.4. Histological Analysis by Hematoxylin and Eosin (H&E) Staining

Tissue samples were collected and fixed in 4% paraformaldehyde at room temperature for 24 h. Subsequently, the samples were dehydrated using a graded ethanol series and embedded in paraffin. The intestinal tissues were sectioned, followed by deparaffinization and rehydration. H&E staining was performed for intestinal tissue analysis.

### 2.5. Quantitative Real‐Time PCR (qRT‐PCR)

Total RNA was extracted from ileum using TRIzol reagent (Invitrogen, 15596018CN) per the manufacturer’s instructions. cDNA was synthesized with HiScript III All‐in‐one RT SuperMix for qRT‐PCR (Vazyme, R333‐01). qRT‐PCR was performed using SYBR Green (Vazyme, Q711‐02) on a QuantStudio 6 Flex instrument. The expression of APOL1 was normalized to *GAPDH*, and the relative expression levels were determined using the 2^−ΔΔCt method.

Primers used:

*GAPDH*‐ Fwd: TCTGACTTCAACAGCGACAC;

Rev: CGTTGTCATACCAGGAAATGAG.

*APOL1* ‐ Fwd: GCTTTGCTGAGAGTCTCTGTCC;

Rev: GGGCTTACTTTGAGGATCTCCAG.

*IL6*‐ Fwd: CCTGAACCTTCCAAAGATGGC;

Rev: TTCACCAGGCAAGTCTCCTCA.

*IL1*β‐ Fwd: TTACAGTGGCAATGAGGATGAC;

Rev: GTCGGAGATTCGTAGCTGGAT.

*TNFa*‐ Fwd: CTCTTCTGCCTGCTGCACTTTG;

Rev: ATGGGCTACAGGCTTGTCACTC.

*CD86*‐ Fwd: CCATCAGCTTGTCTGTTTCATTCC;

Rev: GCTGTAATCCAAGGAATGTGGTC.

*CD206*‐ Fwd: AGCCAACACCAGCTCCTCAAGA;

Rev: CAAAACGCTCGCGCATTGTCCA.

*iNOS*‐ Fwd: AGGTAGAGGCCTGGAAAACC;

Rev: GTCTTGCGCATCAGCATACA.

*NF-κB p65*‐ Fwd: TGAACCGAAACTCTGGCAGCTG;

Rev: CATCAGCTTGCGAAAAGGAGCC.

### 2.6. Plasma APOL1 Quantification by ELISA

Plasma APOL1 concentrations in NEC (Medical NEC = 5 and Surgical NEC = 10) and CTRL (*n* = 15) groups were quantified using a commercial human APOL1 ELISA kit (Shanghai Zhenke Biotech, Cat. ZK‐2519) following the manufacturer’s protocol.

### 2.7. Immunofluorescence Staining of Tissue Sections

Paraffin sections were deparaffinized, rehydrated, and subjected to antigen retrieval with EDTA buffer (pH 9.0). Sections were incubated with 3% hydrogen peroxide for 30 min at room temperature, blocked with 5% goat serum for 1 h, and incubated overnight at 4 °C with APOL1 primary antibody (Proteintech, 11 486‐2‐AP, rabbit, 1:600) and CD86 (Abcam, ab239075, rabbit, 1:100). For multiplex immunofluorescence staining, sections were incubated overnight at 4 °C with primary antibody pairs as follows: APOL1 and CD86: anti‐APOL1 (Proteintech, 11486‐2‐AP, rabbit, 1:600) and anti‐CD86 (Abcam, ab239075, rabbit, 1:100). APOL1 and EpCAM: anti‐APOL1 and anti‐EpCAM (Santa Cruz, sc‐66020, mouse, 1:100). APOL1 and CD31: anti‐APOL1 and anti‐CD31 (Santa Cruz, sc‐376764, mouse, 1: 100). On day 2, HRP–conjugated secondary antibody (Beyotime, P0948, rabbit, 1: 1000) was added and incubated at room temperature for 1 h. Subsequently, TSA working solution was prepared, and the sections were incubated with it in the dark for 10 min to amplify the signal. Finally, nuclei were stained with DAPI. Images were captured and analyzed using a Leica DMi8 microscope.

### 2.8. Cell Culture and Macrophage Polarization

The human monocytic cell line THP‐1 (species: *Homo sapiens*; sex: male; tissue of origin: peripheral blood from a patient with acute monocytic leukemia; official cell line name: THP‐1; RRID: CVCL_0006) was obtained from the Cell Bank of the Chinese Academy of Sciences in June 2024. The cell line was authenticated by short tandem repeat (STR) profiling and has not been previously reported as misidentified or contaminated. Additionally, the cell line was confirmed to be free of mycoplasma contamination using the PCR–based method prior to the described experiments. RPMI‐1640 medium (Gibco, 11875093) was supplemented with 10% fetal bovine serum (FBS, Pricella, 164218) and 1% penicillin/streptomycin (biotech, C100C8) to prepare the complete culture medium. THP‐1 cells were differentiated into M0 macrophages by treatment with 150 nM phorbol 12‐myristate 13‐acetate (PMA, Biosharp, BL1127A) for 24 h. For M1 polarization, cells were treated with 100 ng/mL lipopolysaccharide (LPS, Sigma, L2880) and 20 ng/mL interferon‐γ (IFN‐γ, Novoprotein, C014) for 24 h, with or without the APOL1 inhibitor inaxaplin (VX‐147, Sellcekchem, E1288). For M2 polarization, cells were treated with 20 ng/mL IL‐4 (Novoprotein, CX03) and 20 ng/mL IL‐13 (Novoprotein, CC89) for 48 h.

### 2.9. Flow Cytometric Analysis

The flow cytometry analysis was performed on THP‐1 cells that were first differentiated into M0 macrophages using PMA and then polarized to M1 phenotype (using LPS/IFN‐γ) in the presence or absence of the APOL1 inhibitor VX‐147. Cells were collected by detaching with Accutase. After washing with PBS, the cells were incubated with CD86‐FITC (Elabscience, E‐AB‐F1012C) plus CD206‐APC (Elabscience, E‐AB‐F1161E) for 30 min at 4 °C. Parallel cell samples were loaded with 10 μM 2’,7’‐dichlorodihydrofluorescein diacetate (DCFH‐DA; Beyotime, S0033S) and incubated at 37 °C for 30 min in the dark. Cells were then washed twice with PBS. Viability was assessed by 7‐AAD (Cat No. PD00101). Cells were first gated on FSC‐A vs. SSC‐A to exclude debris. Single cells were selected using FSC‐H vs. FSC‐A gating to exclude doublets. To confirm polarization states, cells were stained for the M1 marker CD86 and M2–associated marker CD206. M1 polarization was specifically quantified as the percentage of CD86 + CD206− cells within the macrophage population. Intracellular ROS levels were measured as the mean fluorescence intensity (MFI) in the FITC channel of the samples. Data were acquired on a FACSCanto and analyzed using FlowJo v10.8.

### 2.10. Statistical Analysis

Transcriptomics and proteomics data were analyzed using R (v4.4.1). For tissue transcriptomics, raw counts were normalized by DESeq2, and differential expression was assessed using the DESeq2 negative binomial model with Benjamini‐Hochberg correction. Detected genes were subjected to Gene Set Enrichment Analysis (GSEA). For plasma proteomics, data were normalized using the DEP package, with missing values imputed via random Gaussian distribution. Differential proteins were identified by |log2FC| > 0.584 and *p* < 0.05 for Gene Ontology‐Biological Process (GO‐BP) enrichment analysis. Experimental data were analyzed and visualized using GraphPad Prism 9.5, with Spearman correlation tests used to assess linear relationships. All *p*‐values were two‐sided, with *p* < 0.05 considered significant.

## 3. Results

### 3.1. APOL1 Expression Was Increased in NEC

Through tissue transcriptomics analysis combined with qPCR validation, we found that the gene transcription levels in the NEC group *APOL1* were significantly upregulated compared with CTRL (Figure 1a,b). Furthermore, plasma proteomics analysis of an cohort (CTRL = 20, Medical NEC = 10, and Surgical NEC = 17) identified a marked elevation in APOL1 expression in NEC patients (Figure 1c, Table S1). This finding was subsequently validated in a separate validation cohort (CTRL = 15, Medical NEC = 5, and Surgical NEC = 10) via plasma ELISA assays (Figure 1d, Table S2). Histopathological analysis of intestinal tissues revealed that the NEC group exhibited typical inflammatory injury characteristics, including massive infiltration of inflammatory cells, destruction of intestinal villus structure, and extensive mucosal necrosis (Figure 1e). Immunofluorescence staining further confirmed that the protein expression intensity of APOL1 in intestinal tissues of the NEC group was markedly enhanced (Figure 1f,g, *p* < 0.009). These experimental findings are collectively confirming the aberrant overexpression of APOL1 in NEC.

Figure 1The expression level of APOL1 was significantly upregulated in both NEC tissue and plasma. (a) Tissue transcriptomics showing increased expression of *APOL1*. TPM (Transcripts Per Million) is a standardized measure of ene expression levels. (CTRL, *n* = 7; Surgical NEC, *n* = 7). (b) qRT‐PCR validation showing increased expression of *APOL1* in NEC tissues. (CTRL, *n* = 5; Surgical NEC, *n* = 4). (c) Plasma proteomics showing increased expression of APOL1. (CTRL, *n* = 20; Medical NEC, *n* = 10; Surgical NEC, *n* = 17). (d) Plasma APOL1 levels measured by ELISA further confirmed that the expression of APOL1 was significantly elevated in the NEC. (CTRL, *n* = 15; Medical NEC, *n* = 5; Surgical NEC, *n* = 10). (e) Representative H&E staining of human infant intestine in CTRL and NEC groups. Scale bar, 200 um. (CTRL, *n* = 5; Surgical NEC, *n* = 5). (f) Immunofluorescence of NEC ileal lesion tissues showing significant deposition of APOL1 in the NEC mucosal lamina propria. (CTRL, *n* = 9; Surgical NEC, *n* = 9). (g) Statistical analysis of the meanfluorescenceIntensity for APOL1. Data were analyzed using nonpaired sample *t*‐test. (CTRL, *n* = 9; Surgical NEC, *n* = 9). CTRL, control group; NEC, necrotizing enterocolitis; qRT‐PCR, quantitative polymerase chain reaction.

**Figure:**
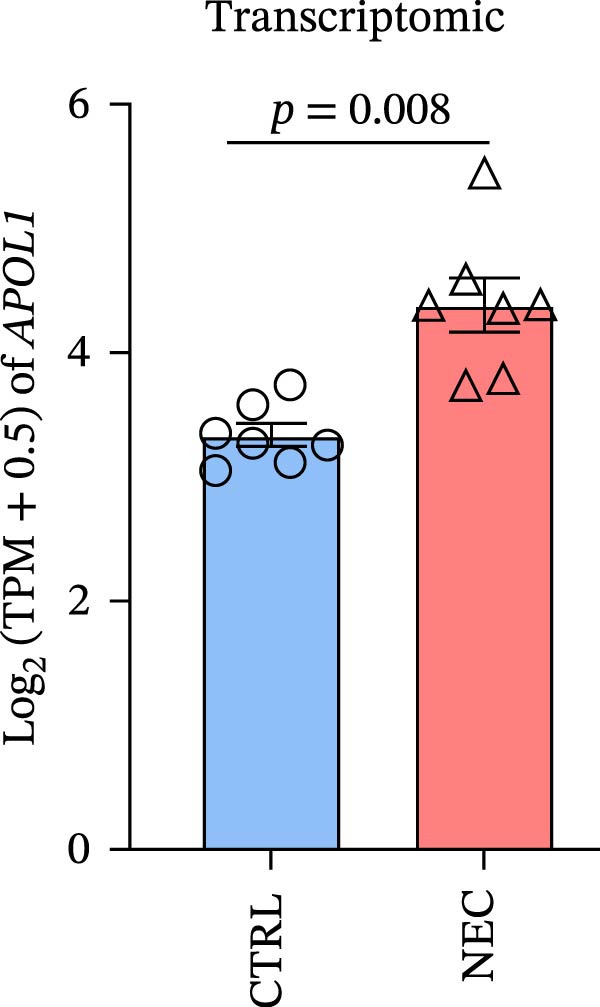
(a)

**Figure:**
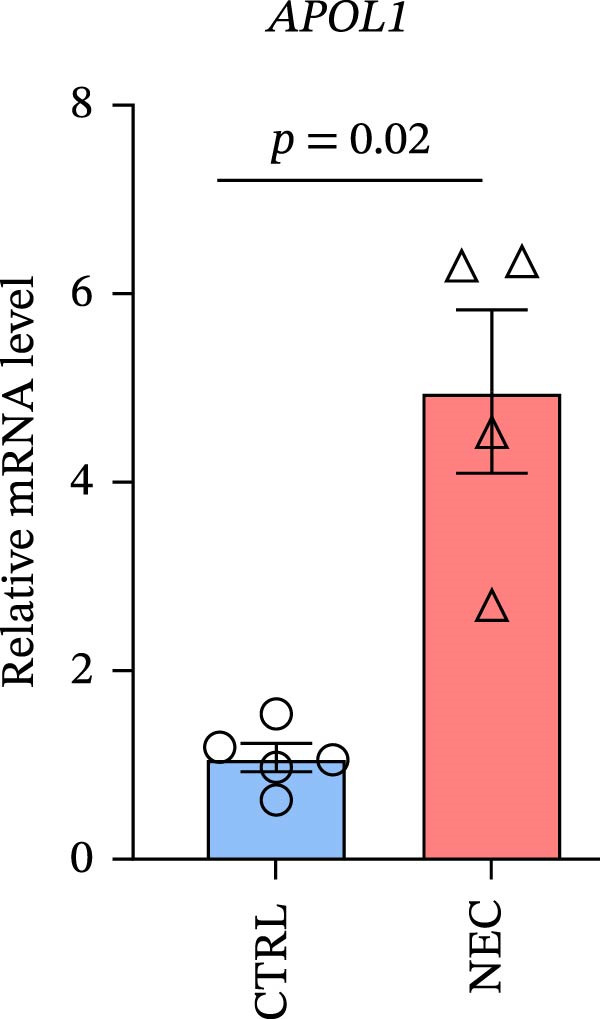
(b)

**Figure:**
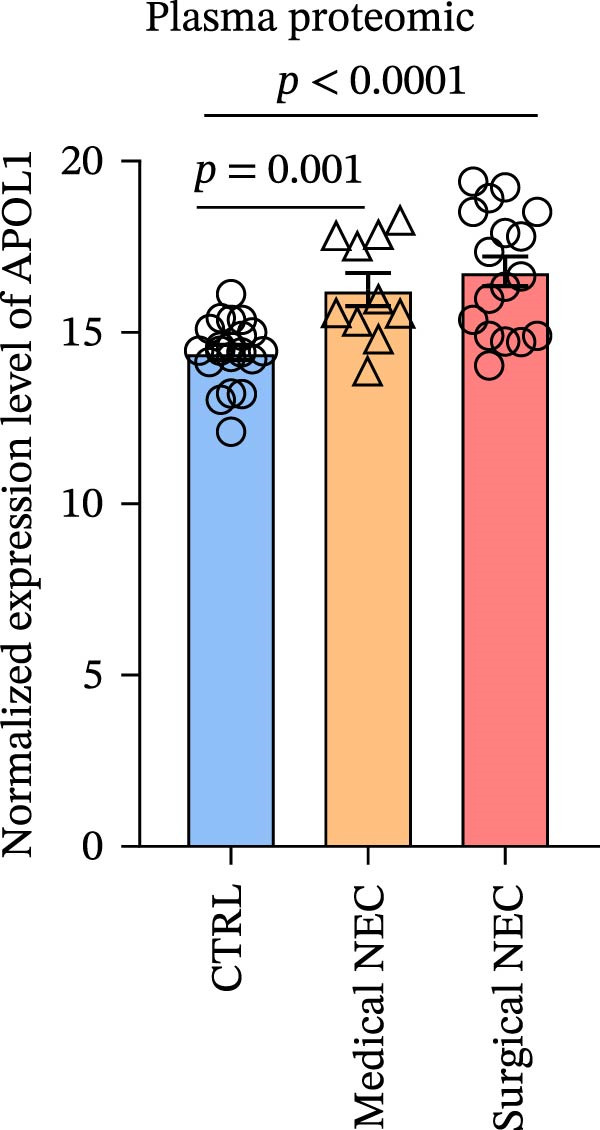
(c)

**Figure:**
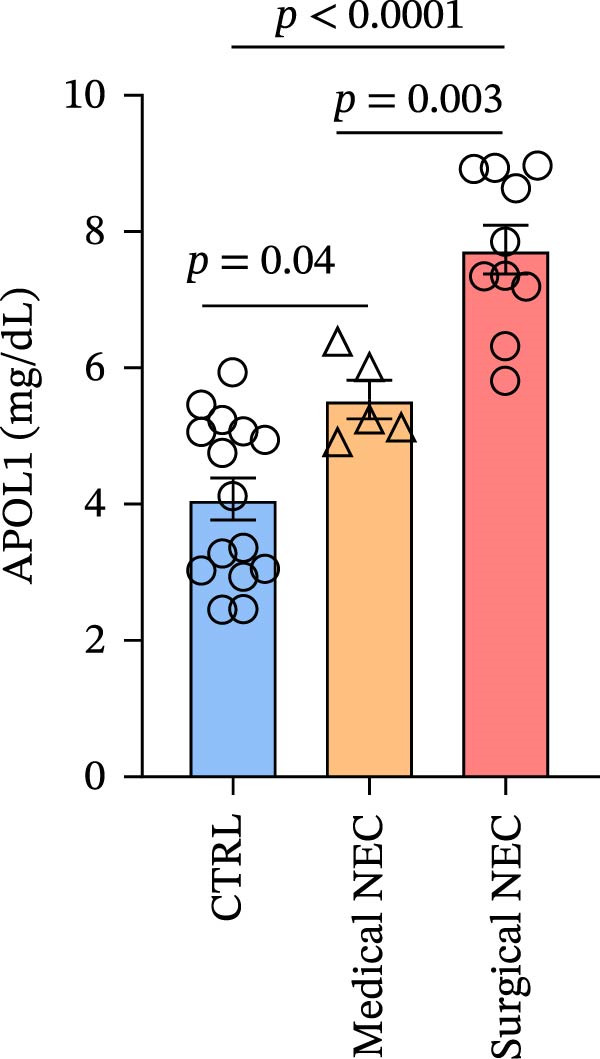
(d)

**Figure:**
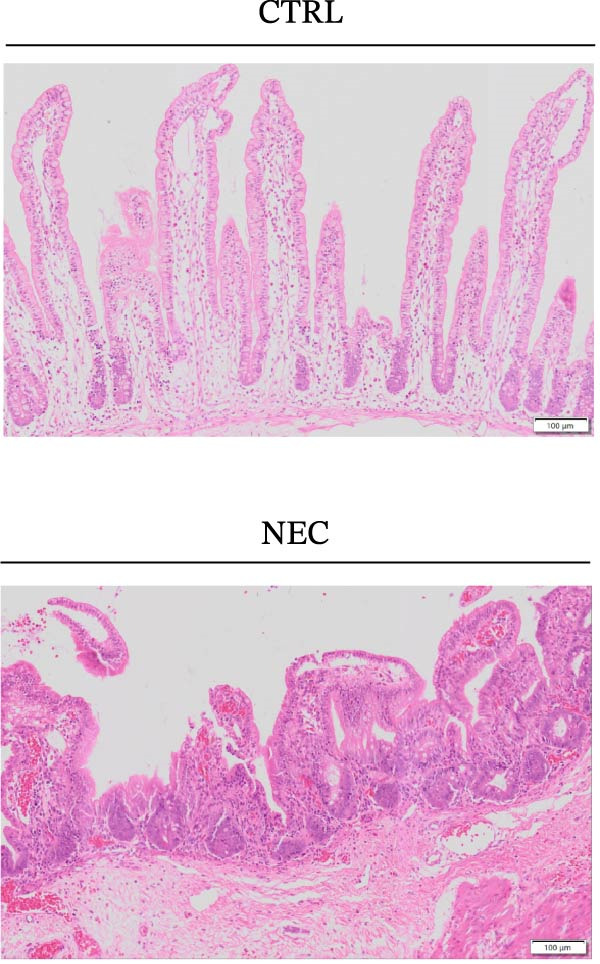
(e)

**Figure:**
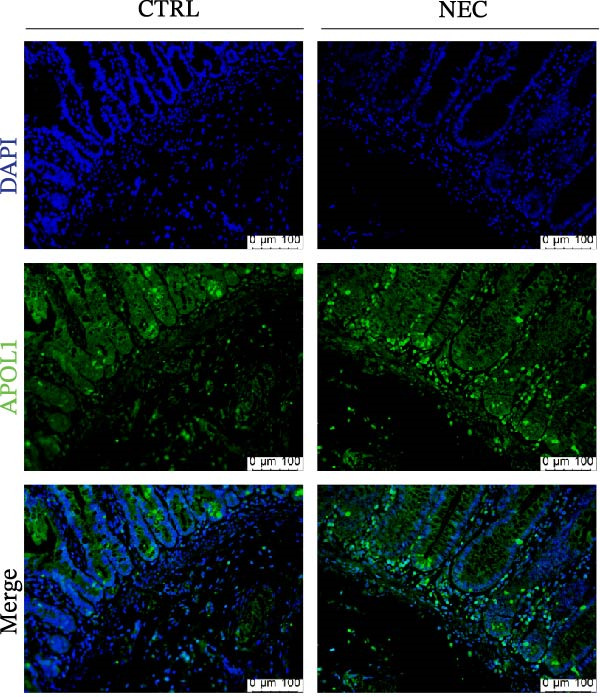
(f)

**Figure:**
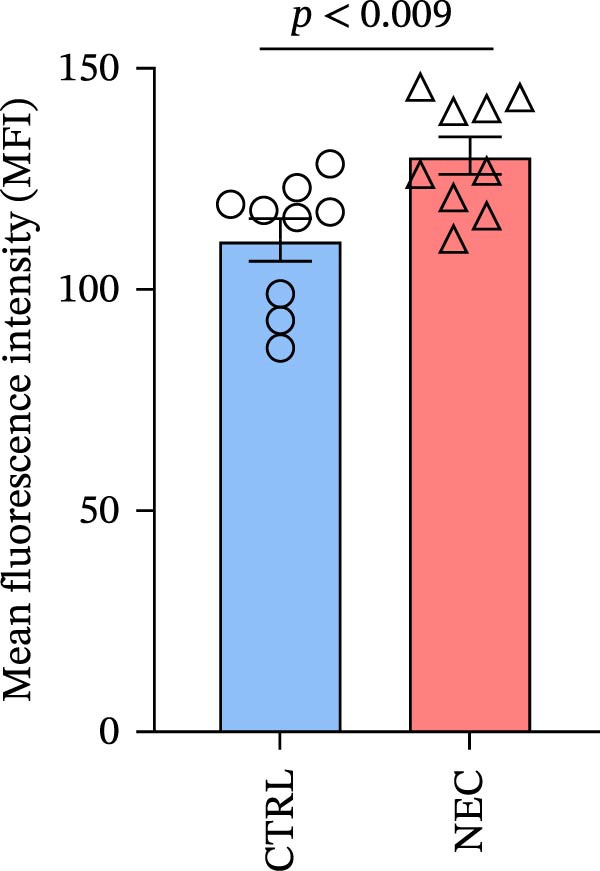
(g)

### 3.2. APOL1–Mediated Cytotoxic Pathway and Macrophage Polarization in NEC

GO‐BP pathway enrichment analysis of peripheral blood plasma proteomics revealed that APOL1 is significantly associated with multiple cytotoxic pathways, including host cell‐mediated killing of pathogens or symbionts (Figure 2a). Consistently, GSEA of the tissue transcriptome showed significant upregulation of these pathways in the NEC group (NES > 1.5) (Figure 2b), suggesting that APOL1 may play a pivotal role in the immune response and inflammatory processes of NEC. To elucidate the effector cell types, we performed immune infiltration analysis, which showed a strong positive correlation between *APOL1* expression and M1 macrophage infiltration (*rs* = 0.72 and *p* = 0.004), while a negative correlation was observed with M2 macrophages (*rs* = ‐0.54 and *p* = 0.045) (Figure 2c). Additionally, *APOL1* expression was positively correlated with proinflammatory cytokines (*IL6*, *IL1*β, and *TNF*) and chemokines (*CCL1*, *CCL2*, *CCL3*, *CCL5*, *CCL8*, *CCL9*, *CCL10*, and *CCL11*) but negatively correlated with the anti‐inflammatory chemokine *CCL14* (Figure 2d). Furthermore, we performed costaining assays for APOL1 and CD86 (a surface marker of M1 macrophages) in intestinal tissues and noted that APOL1 was prominently expressed in areas with elevated CD86 levels (Figure 2e). In contrast, minimal APOL1 signal was detected in areas positive for EpCAM (an epithelial cell marker) or CD31 (an endothelial cell marker) (Figures S1 and S2). This indicates that APOL1 in the inflammatory microenvironment is primarily derived from M1 macrophages. We further validated these findings in the ileum tissues of NEC and CTRL and discovered that the transcriptional levels of *APOL1* were positively correlated with those of *IL6*, *IL1*β, and *TNFα* (Figure 2f). These findings suggest that APOL1 may be involved in NEC–associated inflammatory responses by regulating the polarization of M1 macrophages.

Figure 2APOL1 may promote M1 macrophage polarization and inflammatory cytokine secretion. (a) Gene Ontology‐Biological Process (GO‐BP) enrichment analysis of differential proteins in plasma proteomics. Only the most significant terms involving APOL1 are presented. The circle area reflects the number of proteins on the pathway, and the color depth the −log10 (*p*‐value). (CTRL, *n* = 20; Medical NEC, *n* = 10; Surgical NEC, *n* = 17). (b) Gene Set Enrichment Analysis (GSEA) of all identified genes in tissue transcriptomics highlights significant GO‐BP terms involving *APOL1*. NSE, normalized enrichment score. (c) Immune infiltration analysis in tissue transcriptomics using CIBERSORT. Spearman correlation analysis was performed to assess the association between the expression levels of *APOL1* and immune cell populations. (CTRL, *n* = 7; Surgical NEC, *n* = 7). (d) In tissue transcriptomics, Spearman correlation analysis was conducted to evaluate the association between the expression levels of *APOL1* and those of inflammation‐related cytokines and chemokines. (CTRL, *n* = 7; Surgical NEC, *n* = 7). (e) Immunofluorescence costaining of ileal tissue showed high expression of APOL1 in regions with high CD86 expression. DAPI (blue) marks nuclei, APOL1 (green), and CD86 (red) labels M1 macrophages. Scale bar: 100 μm. (CTRL, *n* = 5; Surgical NEC, *n* = 5). (f) In the ileal tissues of NEC and CTRL, we performed qRT‐PCR validation. Spearman correlation analysis demonstrated that the transcriptional levels of *APOL1* were positively correlated with those of the proinflammatory cytokines *IL6, IL1β*, and *TNFα*. (CTRL, *n* = 4; Surgical NEC, *n* = 4). CTRL, control group; NEC, necrotizing enterocolitis; qRT‐PCR, quantitative polymerase chain reaction; rs, correlation coefficient.

**Figure:**
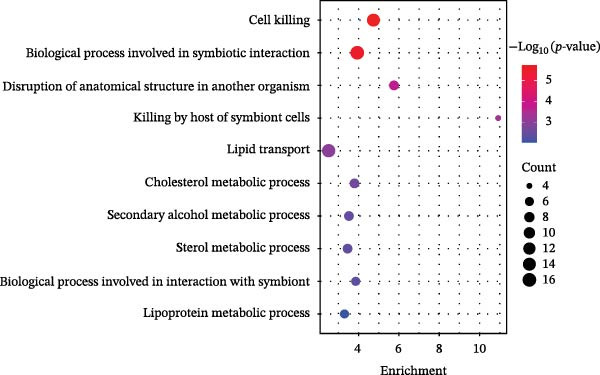
(a)

**Figure:**
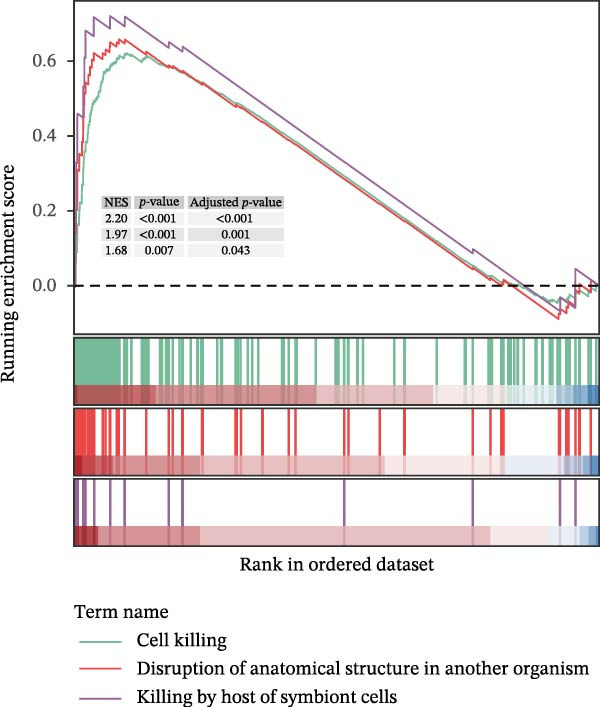
(b)

**Figure:**
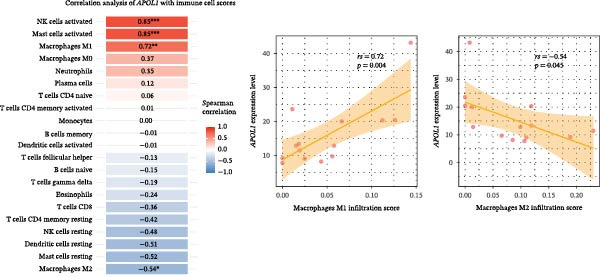
(c)

**Figure:**
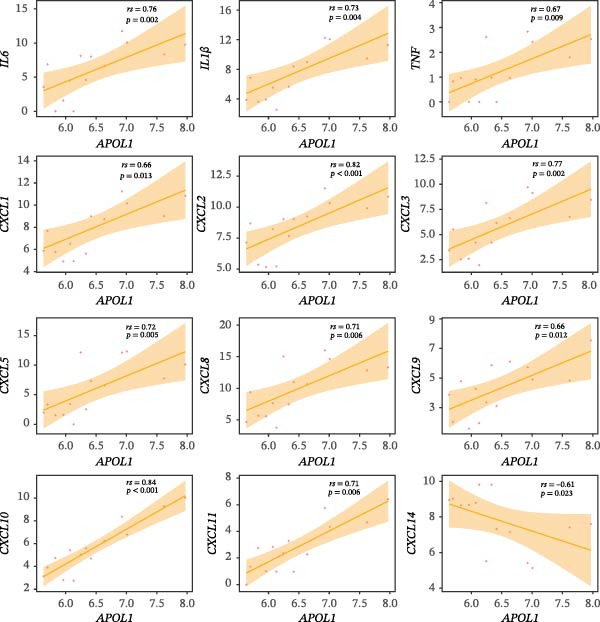
(d)

**Figure:**
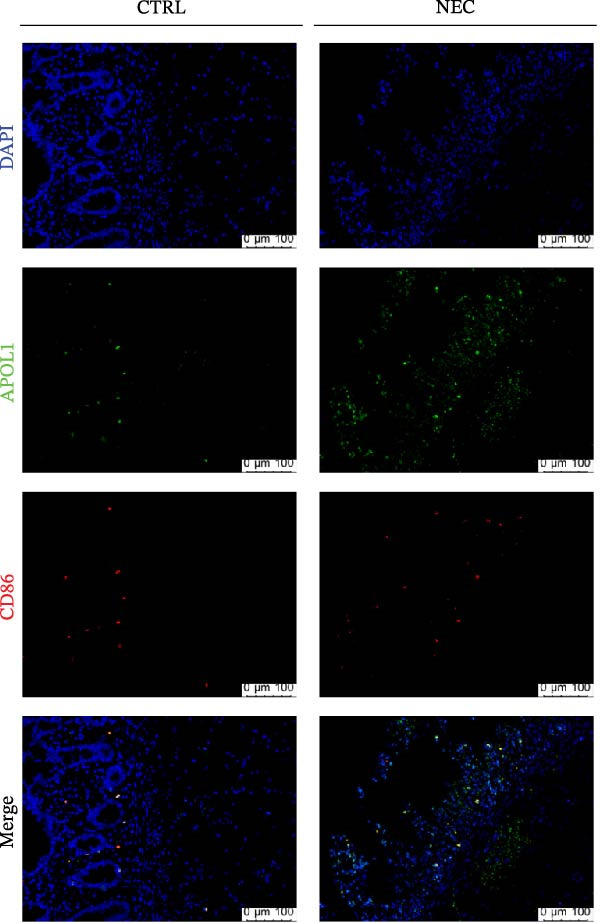
(e)

**Figure:**
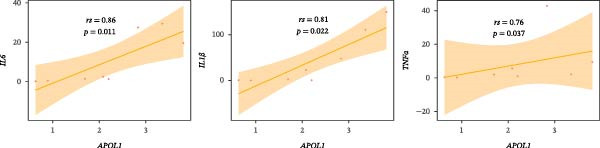
(f)

### 3.3. Inhibition of APOL1 by VX‐147 Attenuates M1 Macrophage Polarization via ROS/NF‐κB Signaling

To further investigate whether APOL1 promotes macrophage polarization toward the M1 phenotype and enhances the secretion of proinflammatory cytokines, we first successfully induced M1 and M2 macrophages from THP‐1 cells (Figure 3a). Notably, *APOL1* was significantly upregulated in M1 macrophages compared to M0 cells, whereas its expression remained unchanged in M2 macrophages (Figure 3b). To validate these findings, we employed VX‐147, a novel small‐molecule inhibitor of APOL1. Immunofluorescence analysis confirmed that VX‐147 treatment effectively reduced APOL1 expression (Figure S3). Consistent with this, VX‐147–treated M1 macrophages exhibited downregulated transcription levels of *CD86*, *IL-6*, *iNOS*, and *NF-κB p65* (Figure 3c). Flow cytometry further demonstrated that VX‐147 treatment suppressed M0‐to‐M1 polarization (Figure 3d). Given that ROS play a critical role in macrophage polarization, particularly by promoting NF‐κB nuclear translocation through IκBα destabilization, thereby upregulating inflammatory genes such as IL‐6 and iNOS, we assessed ROS levels following VX‐147 treatment. Strikingly, VX‐147 significantly reduced ROS production (Figure 3e). These findings demonstrate that APOL1 may promote M1 macrophage polarization and enhance proinflammatory cytokine production via the ROS/NF‐κB signaling axis, thereby contributing to the pathogenesis and progression of NEC.

Figure 3VX‐147–mediated suppression of APOL1 dampens M1 macrophage polarization via attenuation of the ROS/NF‐κB signaling axis. (a) Transcriptional levels of *CD86* and *CD206* during THP‐1 polarization. THP‐1 monocytes were differentiated into M0 (PMA alone), M1 (PMA + LPS/IFN‐γ), or M2 (PMA + IL‐4/IL‐13) macrophages. (b) Transcriptional levels of *APOL1* across THP‐1 polarization states. (c) Transcription levels of *CD86*, *IL-6*, *iNOS*, and *NF-ĸB p65* in M0 macrophages induced to M1 macrophages with or without *APOL1* inhibitor (VX‐147). (d) Flow‐cytometric quantification of CD86^+^CD206^-^ cells in M0 macrophages induced to M1 macrophages with or without VX‐147. (e) Flow‐cytometric quantification of intracellular reactive oxygen species (ROS) in M0 macrophages induced to M1 macrophages with or without VX‐147. MFI, mean fluorescence intensity. Bars represent the mean ± SD of three replicates (*n* = 3).

**Figure:**
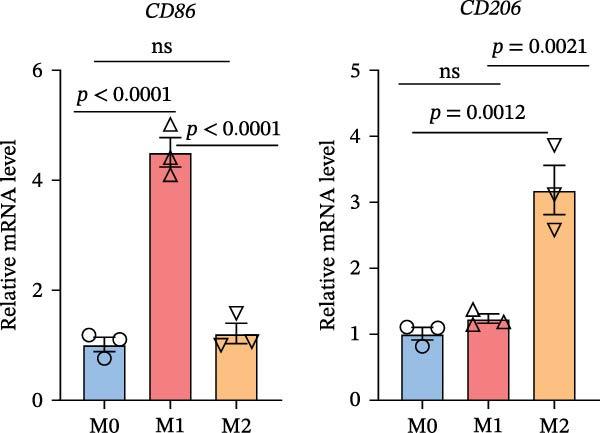
(a)

**Figure:**
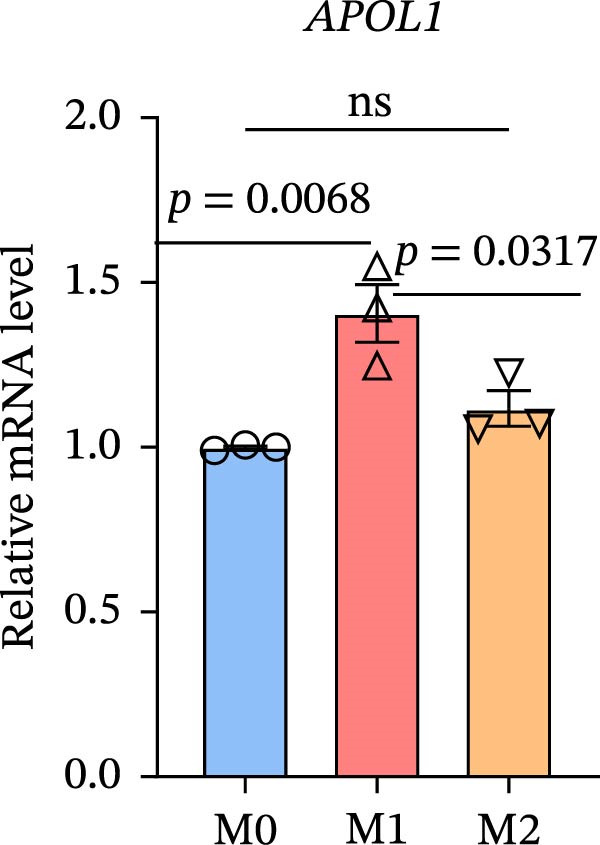
(b)

**Figure:**
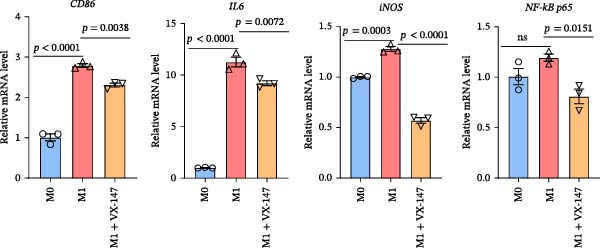
(c)

**Figure:**
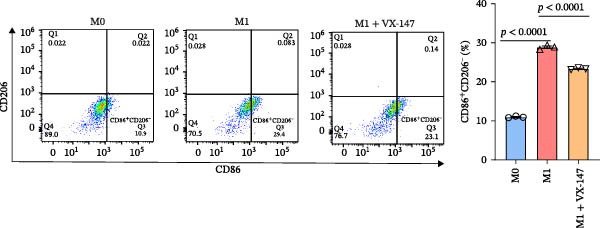
(d)

**Figure:**
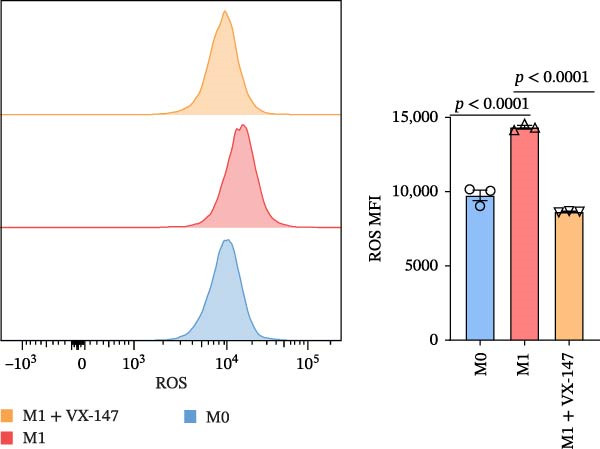
(e)

### 3.4. Evaluation of the Diagnostic Potential of APOL1 in NEC

In the plasma proteomics data (Medical NEC = 10 and Surgical NEC = 17), we found that plasma APOL1 levels were significantly positively correlated with C‐reactive protein (Figure 4a, CRP, *rs* = −0.52, *p* = 0.006) and negatively correlated with platelet count (PLT, *rs* = −0.53, *p* = 0.005) and hemoglobin levels (Hb, *rs* = −0.40, *p* = 0.039) in NEC cases, with stronger correlations in surgical NEC cases (CRP, *rs* = 0.65; PLT, *rs* = −0.57; Hb, *rs* = −0.54). Univariate logistic regression analysis indicated that plasma APOL1 is an independent predictor of NEC (OR = 4, 95% CI 1.61‐9.93, *p* = 0.0028; Figure 4b). ROC curve revealed that plasma APOL1 alone had an AUC of 0.86 for diagnosing NEC (*p* < 0.0001). When combined with lymphocyte count (LYM) or Hb, biomarker APOL1 yields an AUC of 0.91–0.94 for diagnosing NEC and an even higher AUC of 0.95–0.97 for identifying surgical NEC (CTRL = 20, Medical NEC = 10, and Surgical NEC = 17) (Figure 4c). To further validate the diagnostic performance of APOL1, we conducted plasma ELISA assays (CTRL = 15, Medical NEC = 5, and Surgical NEC = 10). Plasma levels of APOL1 yielded an AUC of 0.95 for NEC diagnosis. When combined with LYM, the AUC increased to 0.96, with a high diagnostic performance characterized by both sensitivity and specificity of 93.3% (Figure 4d).

Figure 4Diagnostic value of plasma APOL1 as a biomarker for NEC. (a) Spearman correlation test was used to analyze the correlation between plasma APOL1 expression levels and common hematological clinical indicators in NEC patients. (Medical NEC, *n* = 10; Surgical NEC, *n* = 17). Correlation coefficient (*rs*). (b) Univariate logistic regression analysis showed that plasma APOL1 was significantly associated with NEC, with an odds ratio (OR) of 4 in the NEC group compared to CTRL. (CTRL, *n* = 20; Medical NEC, *n* = 10; Surgical NEC, *n* = 17). (c) Discovery‐cohort plasma proteomics were subjected to receiver operating characteristic (ROC) analyses to assess the diagnostic performance of APOL1 alone and in combination with LYM or Hb for NEC. (CTRL, *n* = 20; Medical NEC, *n* = 10; Surgical NEC, *n* = 17). (d) ROC analysis was applied to ELISA–derived levels in an independent validation cohort to evaluate the diagnostic accuracy of APOL1 alone and in combination with LYM or Hb for NEC. (CTRL, *n* = 15; Medical NEC, *n* = 5; Surgical NEC, *n* = 10). CTRL, control group; CRP, C‐reactive protein; Hb, hemoglobin level; LYM, lymphocyte count; NEC, necrotizing enterocolitis; PLT, platelet count.

**Figure:**
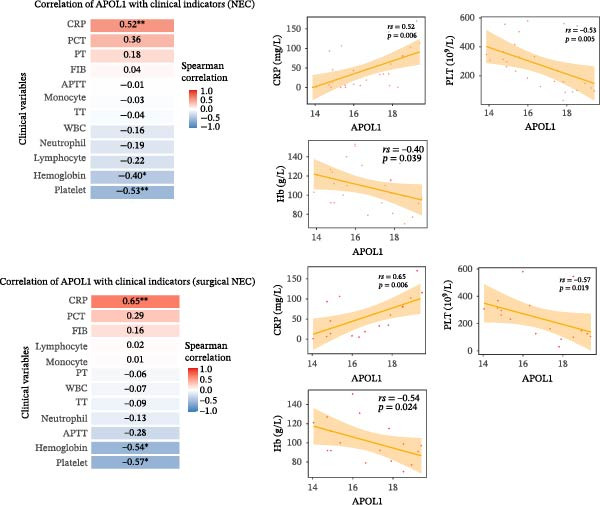
(a)

**Figure:**
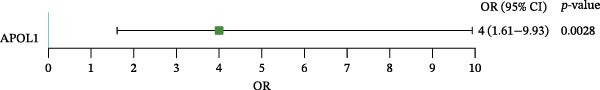
(b)

**Figure:**
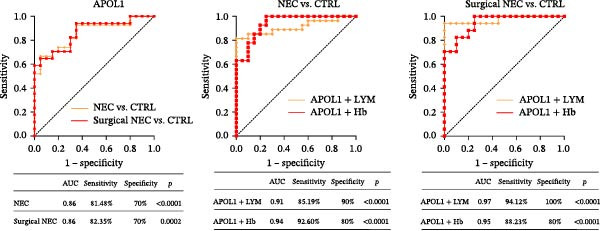
(c)

**Figure:**
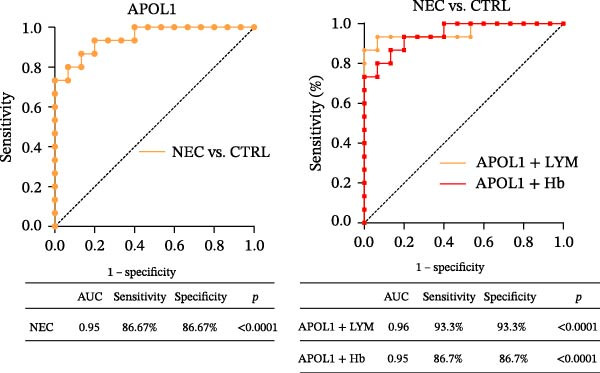
(d)

### 3.5. Proposed Mechanism of APOL1 in Promoting Intestinal Inflammation in NEC via the ROS–NF‐ĸB Axis in M1 Macrophages

**Figure 5:**
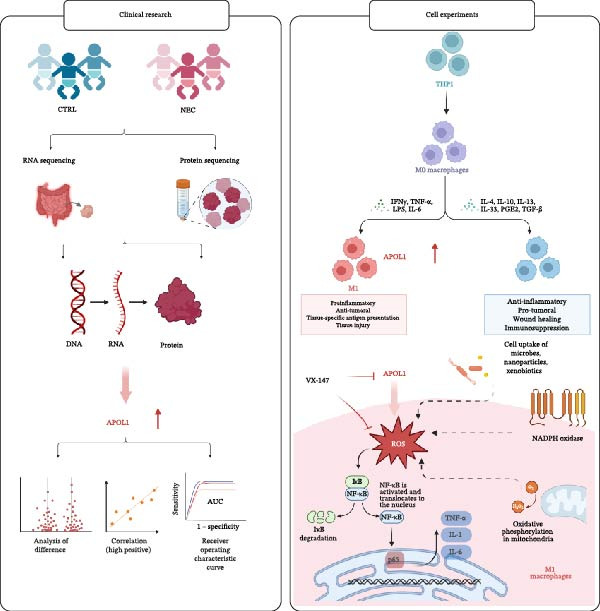
How APOL1 contributes to intestinal inflammation in NEC via the ROS–NF‐κB axis in M1 macrophages: a proposed model.

Tissue transcriptomic and plasma proteomic analyses revealed elevated APOL1 levels in NEC, validated at both RNA and protein levels. In two independent cohorts, plasma APOL1 combined with LYM showed high diagnostic accuracy for NEC. Immune infiltration analyses demonstrated that *APOL1* expression positively correlated with M1/proinflammatory markers and negatively correlated with M2/anti‐inflammatory factors, while in vitro studies demonstrated that inhibition of APOL1 not only downregulated *NF-κB p65* expression and reduced proinflammatory factors (*IL-6* and *iNOS*) in M1 macrophages but also suppressed ROS generation. Given prior reports that the ROS–NF‐ĸB axis is hyperactivated in NEC [15], we hypothesize that APOL1 exacerbates NEC inflammation by potentiating the ROS–NF‐κB signaling axis in M1 macrophages (Figure 5).

## 4. Discussion

NEC is a severe intestinal disease predominantly affecting preterm infants. Our study demonstrates that APOL1 is significantly elevated in NEC at both transcriptional and protein levels, as revealed by integrated multiomics analysis and experimental validation. Notably, APOL1, as an independent risk factor with high diagnostic efficacy, may serve as a novel biomarker for NEC. These findings suggest that APOL1 may drive M1 macrophage polarization by enhancing ROS–dependent NF‐κB activation, thereby exacerbating intestinal inflammation in NEC. Pharmacological inhibition of APOL1 with VX‐147 effectively attenuated this pathogenic cascade.

APOL1 is a secreted high‐density lipoprotein involved in lipid transport and metabolism [11], and its expression is regulated by inflammatory signaling pathways, including interferon (IFN) and LPS. It may resist pathogen invasion through mechanisms such as membrane pore formation and lysosome‐mediated cell death [16, 17]. Dysbiosis is known to contribute to NEC via IFN and LPS metabolic pathways [18, 19]. Our results demonstrate that APOL1 is significantly upregulated in NEC, consistent with findings in other inflammatory diseases [12]. Moreover, NEC has been associated with mitochondrial dysfunction [20] and inflammasome activation [21]. Similarly, APOL1 has been shown to mediate mitochondrial dysfunction [22] and inflammasome activation [23], thereby promoting the progression of various immune‐related inflammatory diseases. Thus, APOL1 may play a critical role in the pathogenesis of NEC.

Macrophages can be polarized into the proinflammatory (M1) and anti‐inflammatory (M2) phenotypes in response to diverse stimuli [24]. NEC is characterized by the accumulation and infiltration of M1 macrophages in the intestinal mucosa [25–27], where activated M1 macrophages secrete proinflammatory cytokines that increase intestinal epithelial cell apoptosis and exacerbate NEC [5, 27]. Restraining M1 polarization while enhancing M2 polarization can safeguard the intestinal mucosa and alleviate intestinal inflammation [28, 29]. Specifically, the initiation of NEC is frequently associated with Gram‐negative Enterobacteriaceae infections. The pathogen‐associated molecular patterns (PAMPs) derived from these bacteria are recognized by host pattern recognition receptors (PRRs), which activate macrophages and drive their polarization towards a proinflammatory phenotype. This pivotal process involves robust ROS generation and potent activation of the NF‐κB pathway, representing a core characteristic of macrophages during infection [30]. In inflammatory states, M1 macrophages secrete CXCL1, CXCL2, and CXCL3 to recruit neutrophils and monocytes, amplifying the inflammatory response [31]. CXCL9, CXCL10, and CXCL11 recruit Th1 cells, natural killer (NK) cells, and other immune cells to the inflamed site through CXCR3 engagement [32], and CXCL10 also promotes M1 polarization [33]. Conversely, M2 macrophages secrete the anti‐inflammatory cytokine CXCL14, which regulates inflammatory balance and induces M2 polarization [34, 35]. In our study, APOL1 expression was significantly elevated at both mRNA and protein levels in NEC–affected tissues. Its expression positively correlated with M1 macrophage infiltration scores and proinflammatory cytokines (*IL6*, *IL1*β, and *TNFa*) and negatively correlated with M2 macrophages and the anti‐inflammatory chemokine *CXCL14*. Further experimental validation revealed high expression of APOL1 in intestinal M1 macrophages. In vitro studies demonstrate that pharmacological inhibition of APOL1 effectively suppresses the ROS/NF‐ĸB signaling pathway, proinflammatory M1 macrophage polarization, and secretion of inflammatory cytokines. Across diverse immune‐mediated disorders, ROS upregulate or activate NF‐ĸB to establish and sustain the proinflammatory M1 macrophage phenotype [36] while enhancing expression of proinflammatory chemokines and cytokines [37]. In NEC, this same ROS–NF‐ĸB axis is hyperactivated, thereby perpetuating a self‐sustaining proinflammatory loop [15]. Therefore, APOL1 may operate as an upstream regulator of ROS–mediated NF‐ĸB activation in macrophages. Inhibitors of APOL1 may hold promise as effective therapeutic agents to suppress the progression of inflammation.

Early clinical manifestations of NEC are nonspecific and often overlap with other conditions such as sepsis and feeding intolerance, leading to delayed diagnosis. Although current imaging techniques and inflammatory biomarkers provide some diagnostic support, their sensitivity and specificity remain insufficient for accurate early detection. Moreover, the rapid progression of NEC and the lack of reliable biomarkers for early risk stratification and disease severity assessment hinder timely clinical decision‐making and the implementation of precision therapies [38, 39]. Fecal calprotectin and S100A12 also show limited diagnostic efficacy, particularly in young children [40, 41]. Near‐infrared spectroscopy is limited by significant interorgan differences in oxygenation, restricting its accuracy [42, 43]. Our analysis revealed that the combination of plasma APOL1 and LYM exhibits high diagnostic efficacy for NEC, with an AUC of 0.96 (sensitivity 93.3% and specificity 93.3%). In NEC, inflammatory markers significantly increase with disease severity, while Hb and PLT decrease due to blood loss and systemic inflammation. Our results indicate that plasma APOL1 expression is positively correlated with CRP levels and negatively correlated with PLT and Hb, with stronger correlations observed in surgical NEC patients (Figure 4a). This further validates the accuracy of APOL1 expression. Moreover, APOL1 may be associated with disease severity.

Several limitations of this study warrant mention. First, given the species‐specific nature of APOL1 expression (predominantly found in primates), existing animal models cannot fully recapitulate the biological functions of human APOL1. Therefore, we were unable to validate its molecular mechanisms through animal experiments. Future studies should employ transgenic technologies or organoid models to elucidate these mechanisms. Second, the single‐center design limits the representativeness of the samples. The diagnostic potential of plasma APOL1 needs to be confirmed through multicenter studies with larger sample sizes.

## 5. Conclusions

In summary, our study demonstrates that APOL1 is highly expressed in NEC and that the APOL1‐ROS‐NF‐κB axis may represent a novel therapeutic target for NEC intervention. Moreover, the combination of plasma APOL1 with LYM or Hb exhibits high diagnostic efficacy for NEC, particularly in surgical cases.

## Author Contributions

All authors contributed to the study conception and design. Jie‐Ting Lu, Qiu‐Hua Wang, Ying‐Yan Liu, and Song Tian contributed to conceptualization, investigation, methodology, validation, visualization, writing of the original draft, review, and editing. Long‐Long Hou, Xin Zhong, Li‐Zhu Chen, and Qian Zhang contributed to conceptualization, data curation, formal analysis, writing of the original draft, review, and editing. Peng‐Fei Wei, Lin Li, Yan Tian, Qiu‐Ming He, Yu‐Feng Liu, and Gen‐Quan Yin contributed to investigation, methodology, resources, software, review, and editing. Yu Ouyang, Lin Liao, Wei Zhong, and Chao‐Ting Lan contributed to conceptualization, investigation, methodology, project administration, resources, supervision, visualization, writing of the original draft, review, and editing.

## Funding

The authors declare financial support was received for the research, authorship, and/or publication of this article. This study was funded by grants from the Science and Technology Project of Guangzhou (Grants 2024A03J1238, 2024A03J1171, and 2025A03J4456), the National Natural Science Foundation of China (Grants 82301955, 82370526, and 82460317), the Basic and Applied Basic Research Foundation of Guangdong Province (Grant 2024A1515013190), the Guangzhou Major Difficult and Rare Diseases Project (Grant 2024MDRD17), and the Guangxi Basic and Applied Basic Research Foundation (Grant 2024JJB140724).

## Disclosure

Chao‐Ting Lan acts as guarantor of the paper, and she takes the responsibility for the integrity of the work as a whole, from its inception to its publication. All authors approved the final version.

## Ethics Statement

This study was performed in line with the principles of the Helsinki Declaration of 1964, as revised in 2000. Ethical approval for this study was obtained from the review board of the Institution Guangzhou Women and Children’s Medical Center (No. 2023307B01). All steps in the study involving human participants were according to the ethical standards of the Institutional Review Board (No. 2023307B01) of Guangzhou Women’s and Children’s Medical Centre, and informed consent was obtained from the parents or legal guardians of the participants. The study was conducted with the Declaration of Helsinki (revised 2013).

## Consent

Participants under 16 years old required parental or legal guardian consent. Written consent for using clinical data and samples was obtained from parents or legal guardians.

## Conflicts of Interest

The authors declare no conflicts of interest.

## Supporting Information

Additional supporting information can be found online in the Supporting Information section.

## Supporting information


**Supporting Information** Table S1. Basic characteristics of the plasma proteomics discovery cohort. Table S2. Basic characteristics of the plasma ELISA validation cohort. Figure. S1.Immunofluorescence costaining of APOL1 with EpCAM in ileal tissue. Figure. S2.Immunofluorescence costaining of APOL1 with CD31 in ileal tissue. Figure. S3. Immunofluorescence assessment of APOL1 expression in M0 macrophages induced to differentiate into M1 macrophages with or without VX‐147.

## Data Availability

All relevant data are contained within the article: The original contributions presented in the study are included in the article, and further inquiries can be directed to the corresponding author.
