# Supplementary material for: The Role of APOL1 in Necrotizing Enterocolitis and Its Promise as a Diagnostic Biomarker
Source: Mediators Inflamm. 2026 Feb 24;2026:8637617. doi: 10.1155/mi/8637617 (PMC12930211; doi:10.1155/mi/8637617)
Supplement: Supplementary file 1 — Supporting Information Table S1. Basic characteristics of the plasma proteomics discovery cohort. Table S2. Basic characteristics of the plasma ELISA validation cohort. Figure. S1.Immunofluorescence costaining of APOL1 with EpCAM in ileal tissue. Figure. S2.Immunofluorescence costaining of APOL1 with CD31 in ileal tissue. Figure. S3. Immunofluorescence assessment of APOL1 expression in M0 macrophages induced to differentiate into M1 macrophages with or without VX‐147. [file MI-2026-8637617-s001.zip › supplementary material.docx]

**supplementary material**

**Supplementary Table**

**Table 1. Basic characteristics of the plasma proteomics discovery cohort.**

| Variables | CTRL  (n=20) | Medical NEC  (n=10) | Surgical NEC  (n=17) |
| --- | --- | --- | --- |
|  |  |  |  |
| Sex (Male) | 12 (60%) | 7 (70%) | 10 (58.8%) |
| Gestational age (week) | 31±2.9 | 30.7±4.3 | 30.7±4.9 |
| Birth weight (kg) | 1.4±0.95 | 1.3±0.14 | 1.5±0.1 |

CTRL, control; NEC, necrotizing enterocolitis.

**Table 2. Basic characteristics of the plasma ELISA validation cohort.**

| Variables | CTRL  (n=15) | Medical NEC  (n=5) | Surgical NEC  (n=10) |
| --- | --- | --- | --- |
|  |  |  |  |
| Sex (Male) | 8 (53.3%) | 3 (60%) | 6 (60%) |
| Gestational age (week) | 31.4±3.2 | 31.1±7.1 | 30.4±4.6 |
| Birth weight (kg) | 1.39±0.07 | 1.28±0.15 | 1.22±0.13 |

CTRL, control; NEC, necrotizing enterocolitis.

**Supplementary Fig.**

**s1**.Immunofluorescence co-staining of APOL1 with EpCAM in ileal tissue. Nuclei were counterstained with DAPI (blue), APOL1 (green), and EpCAM (red) labels intestinal epithelial cells. Scale bar: 100 μm.

**s2**.Immunofluorescence co-staining of APOL1 with CD31 in ileal tissue. Nuclei were counterstained with DAPI (blue), APOL1 (green), and CD31 (red) labels endothelial cells. Scale bar: 100 μm.

**s3**. Immunofluorescence assessment of APOL1 expression in M0 macrophages induced to differentiate into M1 macrophages with or without VX-147. APOL1 (green) and nuclear DAPI (blue).
